# Adaptive responses of *Bacillus cereus* ATCC14579 cells upon exposure to acid conditions involve ATPase activity to maintain their internal pH

**DOI:** 10.1002/mbo3.239

**Published:** 2015-03-05

**Authors:** Khadidja Senouci-Rezkallah, Michel P Jobin, Philippe Schmitt

**Affiliations:** 1 UMR408 Sécurité et Qualité des Produits d'Origine Végétale, INRA, Université d'Avignon84914, Avignon, France; 2 Faculté des Sciences de la Nature et de la Vie, Université de MascaraMascara, Algérie; 3 Département de Microbiologie, Infectiologie et Immunologie, Université de MontréalMontréal, Québec, Canada

**Keywords:** *atpB* gene expression, *Bacillus cereus*, chemostat, F_1_F_0_-ATPase, ionophores, *N,N’*-dicyclohexylcarbodiimide, pH _i_

## Abstract

This study examined the involvement of ATPase activity in the acid tolerance response (ATR) of *Bacillus cereus* ATCC14579 strain. In the current work, *B. cereus* cells were grown in anaerobic chemostat culture at external pH (pH_e_) 7.0 or 5.5 and at a growth rate of 0.2 h^−1^. Population reduction and internal pH (pH_i_) after acid shock at pH 4.0 was examined either with or without ATPase inhibitor *N,N’*-dicyclohexylcarbodiimide (DCCD) and ionophores valinomycin and nigericin. Population reduction after acid shock at pH 4.0 was strongly limited in cells grown at pH 5.5 (acid-adapted cells) compared with cells grown at pH 7.0 (unadapted cells), indicating that *B. cereus* cells grown at low pH_e_ were able to induce a significant ATR and Exercise-induced increase in ATPase activity. However, DCCD and ionophores had a negative effect on the ability of *B*. *cereus* cells to survive and maintain their pH_i_ during acid shock. When acid shock was achieved after DCCD treatment, pH_i_ was markedly dropped in unadapted and acid-adapted cells. The ATPase activity was also significantly inhibited by DCCD and ionophores in acid-adapted cells. Furthermore, transcriptional analysis revealed that *atpB* (ATP beta chain) transcripts was increased in acid-adapted cells compared to unadapted cells before and after acid shock. Our data demonstrate that *B. cereus* is able to induce an ATR during growth at low pH. These adaptations depend on the ATPase activity induction and pH_i_ homeostasis. Our data demonstrate that the ATPase enzyme can be implicated in the cytoplasmic pH regulation and in acid tolerance of *B. cereus* acid-adapted cells.

## Introduction

*Bacillus cereus* is a gram-positive, facultative anaerobe, endospore-forming bacterium that can be isolated from a wide variety of different sites (Kotiranta et al. 2000), and also recognized as one of the major food-borne pathogenic bacteria (McKillip 2000). *Bacillus cereus* is responsible for two types of food-associated illnesses: emetic (vomiting) and diarrheal syndromes. The former is due to a small-molecular-weight cyclic toxin, cereulide, whereas the diarrheal syndrome results from the production of at least two types of multiple-component enterotoxins, hemolysin BL (HBL), nonhemolytic enterotoxin (NHE) (Stenfors Arnesen et al. 2008).

It has been shown that vegetative *B. cereus* cells, like many other bacteria are able to induce an acid tolerance response (ATR) (Thomassin et al. 2006; Desriac et al. 2013). *Bacillus cereus* ATCC14579 and *B. cereus* TZ415 are more tolerant to acid shocks when cells are cultivated at low pH (Jobin et al. 2002; Thomassin et al. 2006). Recently, it has been shown that *B. cereus* ATCC14579 cells can employ complex survival strategies involving decarboxylase and deiminase systems which are implicated in intracellular pH (pH_i_) homeostasis (Senouci-Rezkallah et al. 2011). In response to low pH, Proton pumps play a major role in pH_i_ homeostasis in *Listeria monocytogenes* (Cotter et al. 2000). ATPases from different sources have very similar structures (Santana et al. 1994). They consist of two main subcomplexes: F_1_, the extrinsic membrane subcomplex, which contains the catalytic sites for ATP hydrolysis, and F_0_, the integral membrane subcomplex, which forms the proton channel in bacteria (Kanazawa et al. 1981). In bacteria with a respiratory chain, the primary role of the enzyme is to synthesize ATP from the proton gradient of the respiratory chain. On the other hand, its role is to create a proton gradient (used for a variety of transport processes) with the energy provided by ATP hydrolysis and to maintain the intracellular pH via proton extrusion (Kakinuma 1998), this is the case for the oral streptococci *Streptococcus mutans* and *Streptococcus sanguis* (Bender et al. 1986), *Lactobacillus acidophilus* (Kullen and Klaenhammer 1999) and *Lactococcus lactis* (Koebmann et al. 2000). The proton translocating F_1_F_0_-ATPase enzyme complex plays a significant role in the regulation of intracellular pH in a number of bacteria (Cotter et al. 2000). In *Enterococcus faecalis, Lactobacillus brevis* and *Enterococcus hirae*, a high increase in F_1_F_0_-ATPase activity was observed when cells were grown at low pH (Kobayashi et al. 1984, 1986; Arikado et al. 1999). Little is known about the ATPase activity of *B. cereus*. The enzyme shows similar characteristics as the enzyme isolated from *Escherichia coli* and *B. subtilis* (Banfalvi et al. 1981). The ATPase activity was specifically inhibited by *N,N’*-dicyclohexylcarbodiimide (DCCD) and proton translocation by reacting with the conserved Glu (or Asp) residues of the rotor ring of both F- and V-ATPases (Mizutania et al. 2011). Ionophore antibiotics as valinomycin and nigericin act by specifically increasing the ion permeability of the cell membrane (Bakker 1979). Recently, Mols et al. have demonstrated that genes encoding subunits of the F_1_F_0_-ATPase (*atpB*) were highly downregulated in aerobically grown and exposed *B. cereus* cells upon exposure to sublethal pH 5.4 (Mols et al. 2010a,b; Mols and Abee 2011).

In our study, the role of ATPase activity in ATR and pH_i_ homeostasis of *B. cereus* ATCC14579 was determined. For this goal, anaerobic Chemostat cultures (fermentation) were carried out at constant growth conditions with variation in only one parameter (culture pH). The effect of culture pH, ATPase inhibitor DCCD and ionophores (valinomycin and nigericin) on the ATPase activity, acid survival and thus the internal pH homeostasis of *B. cereus* cells was established.

## Materials and Methods

### Bacterial strains and growth conditions (chemostat culture)

*Bacillus cereus* strain ATCC14579 was obtained from the American Type Culture Collection. Growth medium was J-Broth (JB) (Claus and Berkeley 1986). Chemostat cultures were performed in a 2-L bioreactor (Discovery 100 MRU; INCELETECH, Toulouse, France) using a 1-L working volume. All experiments were carried out at 34°C with agitation at 300 rpm. Culture pH was monitored and maintained at pH 5.5 ± 0.04 or 7.0 ± 0.06. During fermentation, the culture pH was continuously sparged with oxygen-free nitrogen gas to ensure anaerobiosis (Senouci-Rezkallah et al. 2011).

### ATPase activity measurement

A modification of protocol described by (Fortier et al. 2003) was used for cell permeabilization. *Bacillus cereus* cells were grown in a chemostat at different pH values (pH 7.0 or 5.5) and a growth rate of 0.2 h^−1^ (Tourdot-Marechal et al. 1999).

The liberated P_i_ was measured according to (Goffeau and Dufour 1988). ATPase activity was expressed as U mg^−1^ (*μ*mol L^−1^ of P_i_ produced per min and per mg of total protein). Protein concentration was determined using a Bio-Rad (France), protein assay.

The effect of DCCD inhibitor and ionophores on ATPase activity of *B. cereus* cells was investigated by incubation of cells in the presence of DCCD (0.2 mmol L^−1^) at 34°C for 30 min, or by incubation in the presence of 1 *μ*mol L^−1^ valinomycin and/or nigericin for 2 min at room temperature (SigmaAldrich Inc, St. Louis, MO) before the ATP addition.

### Effect of DCCD, valinomycin and/or nigericin on cell survival during acid shock

One milliliter aliquot of steady-state *B. cereus* cells was incubated in the absence and presence of 0.2 or 1 mmol L^−1^ of DCCD for 30 min at 37°C or of 1 *μ*mol L^−1^ valinomycin and/or nigericin (prepared in ethanol at 99%) for 2 min at room temperature. The cells were then diluted in 19 mL of JB at pH 4.0 (acid challenge) or at a pH equivalent to growth pH (control condition) and maintained at 34°C with agitation at 100 rpm. Viability loss was determined by viable counts after 40 min. In control experiment, *B. cereus* cells were preincubated in JB medium in the presence of ethanol before acid shock at pH 4.0. Cells counting was calculated according to the manufacturer's instructions and expressed as colony forming units per mL (CFU mL^−1^) as previously described (Thomassin et al. 2006). The limit of detection was 200 CFU mL^−1^.

### Internal pH measurements

Internal pH (pH_i_) was determined as previously described (Thomassin et al. 2006). *Bacillus cereus* cells were grown in a chemostat at different pH values (pH 7.0 or 5.5) and at growth rate *μ *= 0.2 h^−1^. Steady-state growing cells were preincubated in JB medium at a pH equivalent to growth pH_e_ either with or without of DCCD (0.2 mmol L^−1^ for 30 min at 34°C) (SigmaAldrich Inc, St. Louis, MO), and submitted to acid shock at pH 4.0 in JB medium. The pH_i_ of *B. cereus* cells was measured before and after 10 and 40 min of acid shock.

### mRNA preparation and quantification

Quantification of mRNA was performed by real-time polymerase chain reaction (RT-PCR) as previously described (Duport et al. 2004). To evaluate the reproducibility of the method, three independent RNA samples were analyzed in parallel for three independent cultures performed at pH 7.0 and 5.5. Samples were quantified using Light-Cycler Software version 3.5 (Roche Diagnostics, Meylan, France), standardized for the 16S rRNA, and quantified using the mathematical model established by Pfaffl (2001). Standard deviation was found to be roughly constant for the *atpB* gene coded for the ATPase beta chain (BC5306) (ATPase enzyme). The primers used were F: 5′-GCAATATGTTCGCCAGCTTC-3′, (forward) R: 5′-TCGCAGCTTAGCTCTTCG-3′ (reverse).

### Statistical analysis

Acid stress resistance, pH_i_ and ATPase activity measurements were all determined in triplicate at different times on the same chemostat for each pH tested. The mean value and standard deviation were calculated from the data obtained from the three separate experiments. Results were submitted to variance analysis using Systat 9 software (SPSS, Chicago, IL). Analysis of variance was performed for multiple comparisons of means using Tukey's honestly significant difference test at the 5% level.

## Results

### Effect of growth pH_e_ on level of ATPase

In order to determine the effect of growth pH_e_ on ATPase activity, *B. cereus* cells were grown at pH_e_ 5.5 or 7.0 and at *μ *= 0.2 h^−1^, and the ATPase activity was measured (Table1). The ATPase activity was increased as the growth pH_e_ decreased (9.38 ± 0.31 and 5.21 ± 0.12 U mg^−1^ proteins at pH_e_ 7.0 and 5.5, respectively). We also investigated the effect of DCCD, valinomycin and nigericin on the ATPase activity. In these experimental conditions, DCCD, valinomycin and nigericin were prepared in ethanol at 99%. Hence, we studied the effect of ethanol on the ATPase activity (as control experiment). The results showed that ethanol has no direct effect on ATPase activity of unadapted cells, but it has a visible effect on ATPase activity of acid-adapted cells. For unadapted and acid-adapted cells treated for 30 min with DCCD, ATPase activity was significantly decreased (Table1). These results show that this activity is completely inhibited by DCCD. So this inhibitor had a clear effect on ATPase activity whatever the growth pH.

**Table 1:** Effect of growth pH_e_ and ionophores on ATPase activity of steady-state *Bacillus cereus* ATCC14579 cells grown at a pH_e_ 5.5 or 7.0 and at a growth rate of 0.2 h^−1^

Ionophores	ATPase U^1^ mg^−1^ of proteins	
	pH_e_ (7.0)	pH_e_ (5.5)
None^2^	5.24 ± 0.1	9.38 ± 0.3
Ethanol	5.68 ± 1.18	8.4 ± 0.3
DCCD 0.2 mmol L^−1^ 3	0.70 ± 0.2	0.37 ± 0.1
DCCD 1 mmol L^−1^ 3	0.25 ± 0.1	0.34 ± 0.0
Valinomycin 1 *μ*mol L^−1^ 4	4.39 ± 0.3	1.06 ± 0.2
Nigericin 1 *μ*mol L^−1^ 4	0.8 ± 0.2	0.05 ± 0.06
Valinomycin + nigericin (1 *μ*mol L^−1^ each)^4^	2.26 ± 0.4	1.57 ± 0.02

Inhibitor DCCD, valinomycin and nigericin ionophores tested were dissolved in ethanol (99%). DCCD, *N,N’*-dicyclohexylcarbodiimide.

1 ATPase activity was expressed as *μ*mol L^−1^ of P_i_ produced per min.

2 Control cells in the fermentor without inhibitor,

3 Cells incubated in the presence of DCCD for 30 min at 37°C.

4 Cells incubated in the presence of valinomycin and/or nigericin for 2 min at room temperature.

The effect of ionophores valinomycin and nigericin on the ATPase activity was also studied. A 1 mL aliquot of steady-state *B. cereus* cells were incubated for 2 min in the presence of ionophores: 1 *μ*mol L^−1^ of valinomycin or nigericin, or in the presence of both ionophores for pH_i_ with pH_e_ equilibration (ΔpH = 0). The ATPase activity was measured as described above. The results showed that the presence of valinomycin decreases slightly the ATPase activity in unadapted cells (16%) compared to acid-adapted cells (83%). Although the ATPase activity was inhibited completely by nigericin whatever the growth pH. In the presence of both ionophores, the ATPase activity was inhibited by 75% and 57% in acid-adapted and unadapted cells, respectively. Thus, the presence of valinomycin can decrease the effect of nigericin on ATPase activity of *B. cereus* by pH_i_ and pH_e_ equilibration.

### Effect of DCCD, valinomycin and nigericin on acid survival of acid-adapted cells

**Figure 1:**
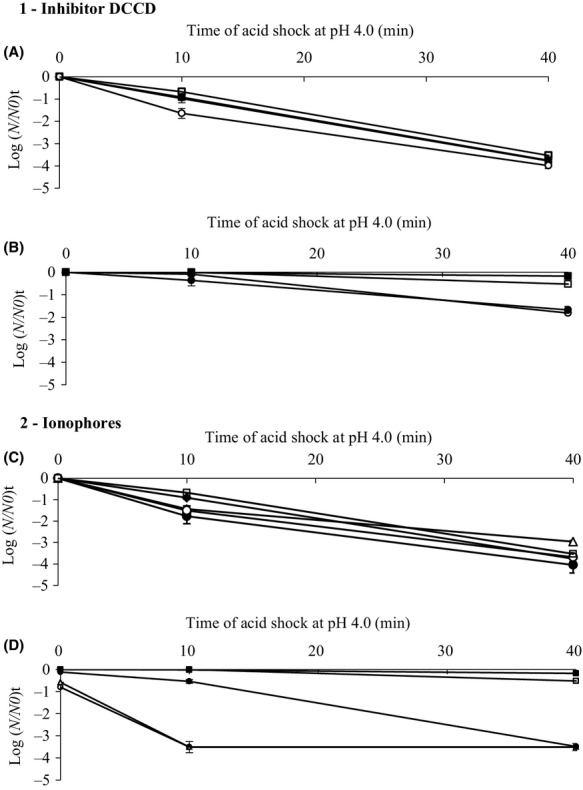
1 – Population decrease during acid shock at pH 4.0 of *Bacillus cereus* ATCC14579 cells from steady-state chemostat cultures grown at growth rate of 0.2 h^−1^ and at pH_e_ 7.0 (A), and pH_e_ 5.5 (B). Prior an acid shock at pH 4.0, the cells were incubated for 30 min without inhibitor at pH 7.0 or 5.5 (▪) as controls and incubated in the presence of ethanol as control experiment (□), with 0.2 mmol L^−1^ *N,N’*-dicyclohexylcarbodiimide (DCCD) (•) or 1 mmol L^−1^ DCCD (○), respectively. 2 – Population reduction during acid shock at pH 4.0 of *B. cereus* ATCC14579 cells from steady-state chemostat cultures grown at growth rate of 0.2 h^−1^ and at pH_e_ 7.0 (C), and pH_e_ 5.5 (D). Prior an acid shock at pH 4.0, the cells were incubated for 2 min without ionophores at pH 7.0 or 5.5 (▪) as controls and incubated in the presence of ethanol as control experiment (□), with 1 *μ*mol L^−1^ valinomycin (•), or 1 *μ*mol L^−1^ nigericin (○) or with 1 *μ*mol L^−1^ valinomycin and nigericin (Δ). *N*_0_ initial population, *N* population after exposure to acid shock at pH 4.0, log(*N/N*_0_) logarithm of population reduction during acid shock at pH 4.0. Data represent the mean values for at least three replicate experiments. Bars represent standard deviation between the 15 experimental data.

In order to investigate whether DCCD had an effect on the acid resistance of *B. cereus*, steady-state cells grown at pH 7.0 or 5.5 were incubated for 30 min in the absence or presence of DCCD (0.2 or 1 mmol L^−1^). In control condition, the population of cells transferred on JB at pH equivalent to growth pH (7.0 or 5.5) in the presence of 1 mmol L^−1^ DCCD was stable (Table2). So, DCCD had no direct effect on *B. cereus* viability. Indeed, acid shock survival of acid-adapted and unadapted cells is not affected by ethanol added for 30 min before acid shock for control experiment (Fig.1). So, ethanol had no major effect on the acid survival of *B. cereus* cells. Pretreatment of *B. cereus* cells with DCCD followed by acid shock at pH 4.0 for 40 min decreased the population about 1.64-log for acid-adapted cells (Fig.1.1B), but had no significant effect on unadapted cells (4-log in absence and presence of DCCD) (Fig.1.1A). Thus, DCCD had clear effect on acid survival of acid-adapted cells. These results indicate that ATPase activity is required for acid adaptation of *B. cereus* cells.

**Table 2:** Effect of DCCD, nigericin and valinomycin on *Bacillus cereus* cells viability

Ionophores	Time of incubation at equivalent pH (min)	Log(*N/N*_0_)_t_	
		7.0	5.5
DCCD 1 mmol L^−1^	10	−0.02 ± 0.01	−0.07 ± 0.11
	40	0.3 ± 0.02	0.7 ± 0.01
Nigericin + valinomycin (1 *μ*mol L^−1^)	10	0.11 ± 0.03	−1.51 ± 0.02
	40	0.12 ± 0.10	−1.51 ± 0.04

Cells from steady-state chemostat cultures grown at a dilution rate of 0.2 h ^−1^ and at pH_e_ 7.0 or 5.5 were transferred on JB at pH identical to the culture pH (7.0 or 5.5) in the presence of 1 mmol L^−1^ DCCD or 1 *μ*mol L^−1^ nigericin and valinomycin instead of the acid challenge were included as controls. Log (*N/N*_0_)_10_ and log (*N/N*_0_)_40_ values are the means of data for the least three replicate experiments. DCCD, *N,N’*-dicyclohexylcarbodiimide.

The effect of ionophores (valinomycin/nigericin) on acid survival of *B. cereus* cells was also studied. Cells grown at pH_e_ 7.0 (unadapted cells) or 5.5 (adapted cells) were incubated for 2 min in the absence and presence of valinomycin or nigericin (1 *μ*mol L^−1^ each), or in the presence of both ionophores and transferred (1) at a pH equal to the initial value (pH 7.0 and 5.5, respectively) as a control condition, or (2) submitted to acid shock at pH 4.0 for 40 min. The corresponding population decrease was determined. The presence of both ionophores at pH_e_ 7.0 (pH_i_ 7.0, ΔpH 0) show no effect on unadapted cells viability. Acid-adapted cells viability decreased by 1.5 log after 10 and 40 min of incubation at pH 5.5 (pH_i_ 5.5, ΔpH 0) (Table2). Thus, the acid resistance of acid-adapted cells was strongly affected by ionophores.

After acid shock, the population decrease of unadapted cells was similar under all four conditions, reaching a value of 4-log after 40 min (Fig.1.2C). Acid-adapted cells preincubated for 2 min in the presence of ionophores presented an initial one log population reduction at the beginning of the acid shock. The population decrease in cells preincubated in the absence of ionophore in pH 5.5 (pH_i_ 6.2, ΔpH 0.72) peaked after 40 min of acid shock 0.2-log reduction compared to cells preincubated for 2 min in the presence of both ionophores at pH 5.5 (pH_i_ 5.5, ΔpH 0) (3.5-log reduction) (Fig.1.2D). After 10 min of acid shock, the population decrease in acid-adapted cells preincubated in the presence of valinomycin is slightly affected compared to the presence of nigericin and both ionophores. Thereafter, the population decrease was similar under all three conditions, reaching a value of 3.5-log after 40 min of acid shock. Thus, the ATPase activity was strongly inhibited by nigericin compared to valinomycin. This suggests that ATPase activity may involved in the acid survival of acid-adapted cells.

### Effect of DCCD on pH_i_ maintenance of *B. cereus* ATCC14579 cells

To examine whether ATPase activity has a role in pH_i_ homeostasis, *B. cereus* cells were grown in a chemostat at pH_e_ 5.5 or 7.0. Steady-state cells were preincubated in the absence or presence of DCCD (0.2 mmol L^−1^) for 30 min, and subjected to acid shock at pH 4.0 for 40 min. The pH_i_ was measured before and during acid shock, and *δ*pH value was calculated.

In unadapted untreated cells by DCCD, pH_i_ was decreased from 7.1 before acid shock to 5.85 and 6.08 after 10 and 40 min of acid shock, respectively (Table3). After preincubation of unadapted cells with DCCD (0.2 mmol L^−1^), pH_i_ was decreased to 4.74 and 4.69 after 10 and 40 min of acid shock, respectively. The pH_i_ of unadapted cells was better maintained in the absence of DCCD.

**Table 3:** Effect of DCCD on internal pH homeostasis and *δ*pH_i_ maintenance of steady-state *Bacillus cereus* ATCC14579 cells grown at pH_e_ 5.5 or 7.0 and at growth rate of 0.2 h^−1^ phi with DCCD

Growth pH_e_	1pH_i_	^a(DCCD 1 mol L−1)^pH_i_	Inhibitor/time of acid shock at pH 4.0 (min)							
			None				DCCD (0.2 mmol L^−1^)			
			2pH_i_	*δ*pH_i_	3pH_i_	*δ*pH_i_	2pH_i_	*δ*pH_i_	3pH_i_	*δ*pH_i_
7.0	7.1 ± 0.12	6.9 ± 0.56	5.85 ± 0.58	1.25	6.08 ± 0.19	1.02	4.74 ± 0.85	2.36	4.69 ± 0.34	2.41
5.5	6.22 ± 0.02	6.15 ± 0.22	5.59 ± 0.52	0.63	5.35 ± 0.4	0.87	5.25 ± 0.04	0.97	4.25 ± 0.24	1.97

Internal pH values were calculated before and after acid shock at pH 4.0 in the absence (control experiment) and presence of 0.2 mmol L^−1^ DCCD. a (DCCD 1 mol L^−1^)pH_i_ before the acid shock incubated in presence of DCCD as negative control. *δ*pH_i_ = pH_i_^a^ − pH_i_^(b or c)^. DCCD, *N,N’*-dicyclohexylcarbodiimide.

1 pH_i_ before the acid shock.

2 pH_i_ after 10 min of acid shock.

3 pH_i_ after 40 min of acid shock.

The pH_i_ of acid-adapted cells was slightly decreased after acid shock and the effect of DCCD was more significant. After 40 min of acid shock, pH_i_ was decreased from 6.22 to 5.35 and 4.25 in absence and in the presence of DCCD (0.2 mmol L^−1^), respectively. These results indicate that the presence of DCCD has an effect on pH_i_ of unadapted cells and this effect was more marked in acid-adapted cells. Thus, activity ATPase inhibition affects the acid survival and pH_i_ maintenance of acid-adapted cells more than unadapted cells.

In addition, we calculated a *δ*pH_i_ value defined as pH_i_ regulation for 40 min of acid shock (pH_i_ after 10 or 40 min of acid shock – initial pH_i_). After 40 min of acid shock, the *δ*pH_i_ was decreased by 1.97 and 2.41 units in *B. cereus* cells treated by DCCD (0.2 mmol L^−1^) compared to untreated cells (*δ*pH_i_ was decreased to 0.87 and 1.02 units) in acid-adapted and unadapted cells, respectively (Table3). So, *δ*pH_i_ was significantly decreased in *B. cereus* cells treated by DCCD after 40 min of acid shock. Thus, internal pH decrease was affected by growth pH and the presence of DCCD. This suggests that the ATPase activity plays a major role in the ATR of *B. cereus* by pH_i_ maintenance.

### Transcriptional analysis

**Figure 2:**
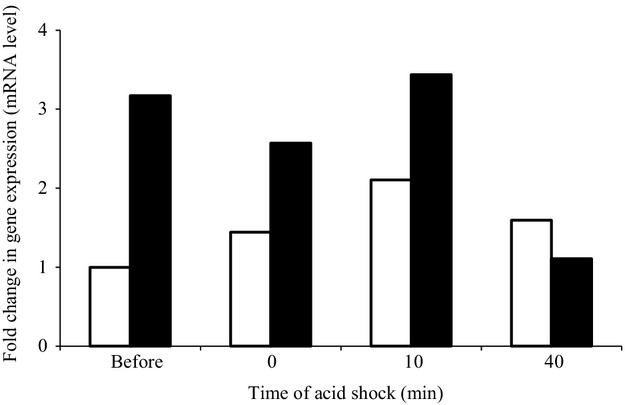
RT-PCR assays conducted on mRNA isolated in steady-state *Bacillus cereus* un-adapted cells (grown at pH 7.0) (□) and acid-adapted cells (grown at pH 5.5) (▪) grown in a chemostat at a growth rate of 0.2 h^−1^. The expression of *atpB* gene before and after 0, 10, and 40 min of acid shock at pH 4.0 was measured. Relative gene expression of *B. cereus* ATCC14579 cells grown at pH 7.0 before acid shock was set at 1.0 (control condition).

RT-PCR assays were conducted to determine the effect of growth pH on ATPase gene expressions. *atpB* (ATP beta chain) transcripts showed a threefold increase in acid-adapted cells grown at pH 5.5 compared with unadapted cells grown at pH 7.0 (Fig.2). After 10 min of acid shock at pH 4.0 *atpB* (ATP beta chain “BC5306”) gene was upregulated in acid-adapted cells (3.5-fold) compared with unadapted cells (twofold). However, we have observed that gene encoding subunits of the F_1_F_0_-ATPase (represented by *atpB*) were highly downregulated in acid-adapted cells (1.6-fold) compared with unadapted cells (onefold) after 40 min of acid shock. Thus the transcription of ATPase gene expression was activated by acid adaptation in *B. cereus*.

## Discussion

*Bacillus cereus* ATCC14579 is able to survive low pH environments. The induced ATR was previously observed in *B. cereus* TZ415 strain (Jobin et al. 2002), NCIMB11796 strain (Browne and Dowds 2002) and ATCC14579 strain (Thomassin et al. 2006), and it was established in other bacteria such as *L. monocytogenes*, *E. coli* and *Salmonella enterica* (O'Hara and Glenn 1994; Davis et al. 1996; Tiwari et al. 2004). *Bacillus cereus* ATCC14579 is able to adapt and to survive the acid stress when it is grown at pH_e_ (5.5) and thus to induce an ATR. These mechanisms of resistance to acid may involve (1) F_1_F_0_-ATPase and/or glutamate or arginine decarboxylases and arginine deiminase, which are involved in intracellular pH (pH_i_) homeostasis (Senouci-Rezkallah et al. 2011), (2) metabolic modifications, and (3) protein synthesis to protect and/or repair macromolecules (Cotter and Hill 2003).

*Bacillus cereus* is a significant acid-resistant neutrophilic bacterium that prefers growth near neutral pH but is able to survive transient exposures to pH 4.0 when preadapted to moderately low pH_e_ (pH 5.5). ATR systems such as ATPase activity may contribute to acid resistance in other bacteria (Mandel et al. 1983; Higuchi et al. 1997; Sakamoto et al. 2002). We observed that the acid shock survival (at pH 4.0) of *B. cereus* cells grown at pH 5.5 (acid- adapted cells) was higher compared to cells grown at 7.0 (unadapted cells). Our results show also that the ATPase activity was increased when cells were grown at low pH 5.5 (acid-adapted cells) compared to cells grown at neutral pH 7.0 (unadapted cells). Similar results were obtained in *Oenococcus oeni* IOB84 when cells were grown at pH 3.5 compared to pH 5.3 (Fortier et al. 2003). This result demonstrate that ATPase activity was increased with pH_e_ decrease. Therefore, if the F_1_F_0_-ATPase is involved in acid adaptation, treatment with DCCD would be expected to render acid-adapted cells more acid sensitive. We found that such treatment just prior to the acid challenge resulted in the significantly enhanced acid sensitivity of the acid-adapted cells during acid shock. So, *B. cereus* population reduction was greatly increased after 40 min of acid shock after DCCD treatment. This reduction was more marked in acid-adapted cells than unadapted cells. Comparable results were observed in *L. monocytogenes* and *Salmonella Typhimurium*, acid-adapted cells grown in batch culture and treated with DCCD were much more sensitive than treated unadapted cells to exposure to pH 3.5 and 3.3, respectively (Foster and Hall 1991; Cotter et al. 2000). These data show the role of F_1_F_0_-ATPase in *B. cereus* acid adaptation.

It is interesting to note that DCCD had no significant effect on unadapted cells at low pH, showing that the ATR is not solely dependent on the activity of this complex. Similarly, our data indicate that the ATPase inhibition by DCCD reached 95% and 96% in cells grown at pH_e_ 7.0 and 5.5, respectively. Previous results showed that both the increased and basal ATPase activity were inhibited equally by DCCD in *Streptococcus faecalis* (Kobayashi et al. 1986). ATPase activity was also significantly inhibited by DCCD in *L. brevis* (Sakamoto et al. 2002). Likewise, in *Lactobacillus* sp. the ATPase activity was also inhibited by DCCD (70% at the concentration of 0.5 mmol L^−1^) (Higuchi et al. 1997). So, the ATPase complex plays a major role in the acid resistance mechanisms in *B. cereus* as previously described in other gram-positive bacteria (Cotter and Hill 2003).

We also observed that ATPase activity was completely inhibited in the presence of nigericin whatever the growth pH. Nevertheless, this activity was inhibited by the valinomycin in acid-adapted cells compared to unadapted cells. Valinomycin renders the plasma membrane permeable to potassium ions, though nigericin exchanges potassium for protons, so that the combined actions of these compounds result in an equilibration of both potassium ions and protons across the membrane and thus the ΔpH. Comparable results were observed in *Lactobacillus* sp. showing that nigericin (2 *μ*mol L^−1^) inhibits completely the ATPase activity, whereas this activity was not inhibited by valinomycin. In the presence of both ionophores, the ATPase activity was inhibited more than 50% in *B. cereus* cells whatever the culture pH. However, the results obtained in *Lactobacillus* sp. show that the activity was completely inhibited (99%) in the presence of valinomycin and nigericin (0.2 *μ*mol L^−1^) (Higuchi et al. 1997). Likewise, it was observed that ATPase activity was inhibited in *Staphylococcus aureus* by DCCD (20 *μ*mol L^−1^) and by nigericin (0.5 *μ*mol L^−1^) (Mandel et al. 1983). Thereafter, we also demonstrate that the population decrease during acid shock of acid-adapted cells is greatly affected by ionophores nigericin and valinomycin. So, our results suggest that ATPase activity can be implicated in the acid survival of *B. cereus*.

To better comprehend *B. cereus* ATR mechanisms at both physiological and genetic levels, it is particularly important to characterize the particular behavior of *B. cereus* in low pH environments. Acid resistance in Gram-positive bacteria involves several strategies, including mechanisms of pH_i_ homeostasis (Cotter and Hill 2003). In *B. cereus*, we have observed that the pH_i_ decreased with growth pH_e_ while maintaining a pH_i_ compatible with cells physiology (Thomassin et al. 2006; Senouci-Rezkallah et al. 2011). This suggested that pH_i_ maintenance may be involved in acid resistance of *B. cereus*. This relatively limited decrease in pH_i_, together with the corresponding increase in ΔpH, could prevent an even more dramatic decline in pH_i_ at lower pH_e_. These results suggest that a mechanism of pH_i_ homeostasis is induced at low pH (pH 5.5). Similar results were observed in *Mycobacterium smegmatis* and *Mycobacterium bovis* BCG, and the lethal pH_i_ for both strains was less than pH 6.0 (Rao et al. 2001). Browne and Dowds confirmed a similar adaptation of *B. cereus* NCIMB11796 in nonregulated batch cultures, where cells were found to maintain their pH_i_ at a higher level than the external acid pH_e_ (Browne and Dowds 2002). Comparable results demonstrate that pH_i_ regulation is involved in acid resistance in *B. cereus* ATCC14579 (Senouci-Rezkallah et al. 2011). We also showed that the pH_i_ values in *B. cereus* acid-adapted and unadapted cells were greatly decreased by the DCCD treatment. Whereas, the *δ*pH value of acid-adapted cells treated by DCCD is not the one observed in unadapted cells after acid shock. This suggests that other mechanisms of pH_i_ homeostasis were induced in acid-adapted. Conversely, it was shown that the pH_i_ of *M. bovis* BCG adapted cells grown at pH 5,0 is decreased in the presence of DCCD, but not affected in cells grown at neutral pH (7.0) (Rao et al. 2001). Thus, our data indicate that F_1_F_0_-ATPase activity plays a major role in acid resistance of acid-adapted cells of *B. cereus*, suggesting that this enzyme may be involved in pH_i_ maintenance. Therefore, ATPase activity may be involved in pH_i_ homeostasis, *δ*pH maintenance and acid resistance in *B. cereus* acid-adapted cells. Clearly, the increase in proton translocation by ATPase activity could enhance the ability of *B. cereus* cells to maintain their pH_i_ and their acid resistance. Thomassin et al. (2006) showed that ΔpH abolition may not allow essential metabolic activities and/or the activity of proteins that are essential for ATR (such as F_1_F_0_-ATPase). The permeability of the cytoplasmic membrane to protons and proton extrusion by F_1_F_0_-ATPase has been established as essential for pH_i_ maintenance in mycobacteria (Rao et al. 2001). Our results show that the ATPase activity in unadapted *B. cereus* cells appears to increase the ATR. In acid-adapted cells, this system may be induced before and during acid shock. We establish therefore that pH_i_ maintenance at low pH by ATPase activity is important to *B. cereus* ATCC14579 ATR through the protons extrusion via ATP hydrolysis.

Since *B. cereus* is a facultative anaerobic bacterium, it could be supposed that this bacterium may use both ATP hydrolysis and synthesis to maintain its pH homeostasis as shown in *L. monocytogenes* (Desriac et al. 2013). The low pH induces the expression of ATPase operon in *B. cereus*, since *atpB* mRNA was upregulated (threefold) in acid-adapted cells compared to unadapted cells. The over-expression of *atpB* gene was also observed after 10 min of acid shock at pH 4.0. Similar results of pH-dependent increase in ATPase transcription were observed in *S. mutans*, *L. monocytogenes*, *L. acidophilus* and *O. oeni* (Kullen and Klaenhammer 1999; Cotter et al. 2000; Quivey et al. 2001). In fact, F_1_F_0_-ATPase encoding genes were downregulated in *B. cereus* ATCC14579 and *B. cereus* ATCC10987 exposed to nonlethal acid conditions, and were not repressed upon exposure to lethal acid stresses, indicating that *B. cereus* does not use F_1_F_0_-ATPase to extrude proton in aerobic conditions (Mols et al. 2010a,b). Genes encoding subunits of the F_1_F_0_-ATPase were highly upregulated in anaerobically grown in a chemostat culture and exposed *B. cereus* cells upon exposure to sublethal pH_e_. Conversely, *B. cereus* does not use F_1_F_0_-ATPase to pump protons out of the cell in aerobic acid conditions and by repressing F_1_F_0_-ATPase genes and lowering the amount of active ATPase, the influx of protons is limited (Mols et al. 2010a,b; Mols and Abee 2011). These results suggest that *B. cereus* ATCC14579 is able to modify *atp* expression and ATPase activity as response to cytoplasmic pH variations and aerobic or anaerobic condition.

Furthermore, downregulation of F_1_F_0_-ATPase genes could be explained by the cells trying to prevent excessive inward flux of proton via this ATPase upon exposure to acid conditions (Mols et al. 2010b). This downregulation has also been demonstrated in *S. aureus* where the expression of F_1_F_0_-ATPase encoding genes was clearly reduced to about 50% (Bore et al. 2007).

We have also demonstrated that *atpB* mRNA was downregulated after 40 min of acid shock. Downregulation of F_1_F_0_-ATPase is best described either by the population reduction or by the translation of mRNA upon exposure to acid conditions. The ATPase could still play an important role by pumping H+ out of the cells, such as Arikado et al. (1999) suggest that the enzyme regulation happens mostly at the posttranscriptional level. It was also shown that the regulation of the enzyme level of F_1_F_0_-ATPase by the intracellular pH, in *S. faecalis*, is mainly at the step of enzyme assembly from its subunits. Consequently, F_1_F_0_-ATPase and antiporters gene regulations under lethal and nonlethal conditions in *B. cereus* cells showed a good equilibrium between ATP synthesis on one hand and proton pumps regulating pH_i_ at the expense of ATP on the other hand (Mols and Abee 2011).

In summary, our study shows that *B. cereus* is able to survive under acid conditions, because it can develop acid survival strategies involving ATPase activity to face severe acid stress. The ATPase activity may protect it against severe acid stress in two ways. Inducing protons extrusion via ATP hydrolysis would produce a less acidic internal pH and generate a positive ΔpH that could help repel protons. This system is of great importance in ATR-induced in *B. cereus*. Complementary work in progress is to construct the ATPase mutant in order to confirm the ATPase activity implication in ATR and pH_i_ maintain of *B. cereus* ATCC14579.
