# Adaptive responses of *Bacillus cereus* ATCC14579 cells upon exposure to acid conditions involve ATPase activity to maintain their internal pH

**DOI:** 10.1002/mbo3.317

**Published:** 2015-12-13

**Authors:** Khadidja Senouci‐Rezkallah, Michel P. Jobin, Philippe Schmitt

**Affiliations:** ^1^ UMR408 Sécurité et Qualité des Produits d'Origine VégétaleINRAUniversité d'AvignonAvignonFrance; ^2^ Faculté des Sciences de la Nature et de la VieUniversité de MascaraMascaraAlgérie

The article entitled “Adaptive responses of *Bacillus cereus* ATCC14579 cells upon exposure to acid conditions involve ATPase activity to maintain their internal pH” published in *MicrobiologyOpen* (Volume 4, Issue 2, pages 313–322, April 2015) incorrectly listed Département de Microbiologie, Infectiologie et Immunologie, Université de Montréal, Montréal, Québec, Canada as one of Khadidja Senouci‐Rezkallah's affiliations. The corrected author information is as follows:

Khadidja Senouci‐Rezkallah^1,2,^*, Michel P. Jobin^1^ and Philippe Schmitt^1^

^1^UMR408 Sécurité et Qualité des Produits d'Origine Végétale, INRA, Université d'Avignon, Avignon, France

^2^Faculté des Sciences de la Nature et de la Vie, Université de Mascara, Mascara, Algérie

*Correspondence

Khadidja Senouci‐Rezkallah, Faculté des Sciences de la Nature et de la Vie, Université de Mascara, Mascara, Algérie Bp 305. E‐mail: khadidja.senouci@icloud.com

